# Unveiling the Enigma: Exploring Risk Factors and Mechanisms for Psychotic Symptoms in Alzheimer’s Disease through Electronic Medical Records with Deep Learning Models

**DOI:** 10.3390/ph16070911

**Published:** 2023-06-21

**Authors:** Peihao Fan, Oshin Miranda, Xiguang Qi, Julia Kofler, Robert A. Sweet, Lirong Wang

**Affiliations:** 1 Computational Chemical Genomics Screening Center, Department of Pharmaceutical Sciences, School of Pharmacy, University of Pittsburgh, Pittsburgh, PA 15213, USA; pef14@pitt.edu (P.F.); osm7@pitt.edu (O.M.); xiq24@pitt.edu (X.Q.); 2 Department of Pathology, Division of Neuropathology, UPMC Presbyterian Hospital, Pittsburgh, PA 15213, USA; koflerjk@upmc.edu; 3 Department of Psychiatry, School of Medicine, University of Pittsburgh, Pittsburgh, PA 15213, USA; sweetra@upmc.edu; 4 Department of Neurology, School of Medicine, University of Pittsburgh, Pittsburgh, PA 15213, USA

**Keywords:** Alzheimer’s disease, dementia, psychosis, deep learning, electronic medical records, comorbidity

## Abstract

Around 50% of patients with Alzheimer’s disease (AD) may experience psychotic symptoms after onset, resulting in a subtype of AD known as psychosis in AD (AD + P). This subtype is characterized by more rapid cognitive decline compared to AD patients without psychosis. Therefore, there is a great need to identify risk factors for the development of AD + P and explore potential treatment options. In this study, we enhanced our deep learning model, DeepBiomarker, to predict the onset of psychosis in AD utilizing data from electronic medical records (EMRs). The model demonstrated superior predictive capacity with an AUC (area under curve) of 0.907, significantly surpassing conventional risk prediction models. Utilizing a perturbation-based method, we identified key features from multiple medications, comorbidities, and abnormal laboratory tests, which notably influenced the prediction outcomes. Our findings demonstrated substantial agreement with existing studies, underscoring the vital role of metabolic syndrome, inflammation, and liver function pathways in AD + P. Importantly, the DeepBiomarker model not only offers a precise prediction of AD + P onset but also provides mechanistic understanding, potentially informing the development of innovative treatments. With additional validation, this approach could significantly contribute to early detection and prevention strategies for AD + P, thereby improving patient outcomes and quality of life.

## 1. Introduction

Alzheimer’s Disease (AD) is the most common neurodegenerative disease affecting 50 million people worldwide [1]. Its presence is associated with a substantial decline in the quality of life [2]. It is estimated that the cost of AD is $604 billion worldwide per year and will triple by the year 2050 [3]. Psychosis, defined by the occurrence of delusions and/or hallucinations, is observed as a common complication of AD. Approximately 50% of patients are likely to suffer from psychotic symptoms after the onset of AD (AD with psychosis, or AD + P) [4]. AD + P patients have more severe cognitive impairments and a quicker cognitive decline than AD patients without psychosis (AD-P) [5,6]. AD + P is also associated with higher rates of co-occurring agitation, aggression, and depression compared to AD-P [5]. These non-cognitive symptoms create burdens not only for people with AD or other dementias but also for their caregivers and are associated with poor outcomes in terms of function, quality of life, disease course, mortality, and economic cost [7,8,9].

The occurrence of psychosis in AD has been shown to be familial, with an estimated heritability of 61%. This suggests that it arises from distinctive biology that could be effectively targeted pharmacologically [10]. More recent studies have helped to elucidate some of the underlying biologic risks of psychosis. A small proportion of the risk is conferred by a greater burden of hyperphosphorylated tau, one of the hallmark pathologies of Alzheimer’s disease. Other pathologies that are often comorbid in AD, such as Lewy bodies, TDP-43 inclusions, and vascular lesions, show less consistent and/or smaller contributions to the risk of psychosis in AD patients [11]. More recent data have highlighted a role for the excess vulnerability of excitatory neurons and synapses in psychosis risk in AD [11,12,13]. 

The use of pharmacotherapy-based treatment options for AD + P has been limited [14,15,16]. Currently, the Food and Drug Administration (FDA) has not approved any specific medication for AD + P. Second-generation antipsychotics (SGAs) are frequently employed and endorsed by geriatric specialists for managing AD + P. However, their application is significantly constrained due to an elevated risk of adverse events and the potential for co-existing health conditions. This led the FDA to issue a “black-box” warning in 2005 to emphasize the heightened risk of mortality for dementia patients receiving SGAs [17]. Simultaneously, antipsychotics have shown only moderate effectiveness in managing psychosis, aggression, and agitation in individuals with dementia. Furthermore, the accelerated cognitive decline present in AD + P (relative to AD without psychosis) is present years before the onset of AD and of psychosis [18], suggesting that there is a window of opportunity to intervene if AD + P can be accurately predicted and if preventative treatments can be identified. This emphasizes the urgent need to discover and develop more effective and safer therapeutic alternatives for AD + P and or agents that may prevent its development [19].

Drawing from our previous research, the limited effectiveness of antipsychotics in treating AD + P can be attributed to their failure to effectively target the underlying biology of the condition [20]. Another study performed a large genome-wide association meta-analysis on 12,317 AD subjects with or without psychosis [21]. The authors reported that AD + P is not significantly genetically correlated with schizophrenia, but it is negatively correlated with bipolar disorder and positively correlated with depression. These findings serve as a reminder to broaden our horizons in search of better treatment options for AD + P.

Deep Neural Networks (DNNs) represent the pinnacle of current machine learning and big data analytics, with a wide array of applications that span from defense and surveillance to human–computer interaction and question-answering systems [22]. DNN architectures can be further categorized into more subtypes for different tasks and data types, including Feed-forward Neural Networks, Convolution Neural Networks (CNNs), and Recurrent Neural Networks (RNNs) [23]. Recently, self-attention-based DNN architecture, transformers, and their variations have been reported to have better model performance than other DNNs [24]. The integration of deep learning in healthcare offers physicians precise disease analysis, leading to improved treatment strategies and, consequently, enhanced medical decision-making. By incorporating deep learning technologies into hospital management information systems, multiple benefits can be achieved: reduction in costs, minimization of hospital stays and their duration, prevention of insurance fraud, detection of changes in disease patterns, improvement in healthcare quality, and more efficient allocation of medical resources [25]. 

Deep learning/data mining algorithms can translate data into information for hypothesis generation through deep hierarchical feature construction to capture long-range dependencies in EMR data. Recently, a variety of deep learning techniques and frameworks have been applied to information extraction, representation learning, outcome prediction, phenotyping, and de-identification [26,27,28,29,30] and yielded better performance than traditional methods and required less time-consuming preprocessing and feature engineering. Specifically, deep learning techniques learn optimal features directly from the data itself, without any human guidance, allowing for the automatic discovery of latent data relationships that might otherwise be unknown or hidden [31]. The proposed research aims to identify potential therapeutic drugs for preventing, delaying, or treating AD + P through an examination of the EMR of AD patients. We predict that drugs capable of minimizing the risk of psychosis development would be beneficial for AD + P management. The deep learning model’s capacity to discern intricate patterns among a multitude of longitudinal features provides us with a comprehensive view of how these variables intersect with AD + P [32]. This, in turn, facilitates the identification of possible novel therapeutic options.

In our previous study, we built a deep-learning-based model, DeepBiomarker, through modification of an established deep-learning framework, Pytorch_EHR [33,34]. In DeepBiomarker, we used diagnosis, medication use, and lab tests as the input, implemented data augmentation technologies to improve the model performance, and also integrated a perturbation-based approach [35] for risk factor identification. In this study, we showed the application of DeepBiomarker on the prediction of AD + P in AD patients for risk/beneficial factor identifications. Compared to the previous version, we have updated the calculation of relative contribution for feature importance. 

## 2. Results

### 2.1. The Performance of DeepBiomarker in AD + P Patients

We have identified 16,294 AD patients from the University of Pittsburgh Medical Center (UPMC) EMR data. We further identified 3535 cases of patients who developed AD + P and 3535 controls who did not develop AD + P within 3 months after the index dates, and all of whom had more than 1 year of EMR data before the index dates. Among the 3535 cases, the average age is 81.9 years old with a standard deviation of 9.06, and the average for the control group is 83.9 years old with a standard deviation of 8.26. There are 2368 female and 1167 male patients in the case cohort and 2333 female/1202 male in the control cohort. The index date for each individual was the date of any encounter after the diagnosis of AD but before the diagnosis of AD + P. Those samples were split into an 8:1:1 ratio for training, validation, and test sets. The validation datasets were used to optimize the parameters in the DeepBiomarker model, and the test datasets were used to evaluate the performance of the established models. The performance of the DeepBiomarker can be found in Table 1. The higher AUC (area under the receiver operating characteristic curve) indicates that the model demonstrates greater accuracy in making accurate predictions. We can see that the two deep-learning-based models implemented in DeepBiomarker, TLSTM, and RETAIN, both achieved high accuracy with an AUC above 0.90 in the validation dataset and test dataset, while the traditional machine learning approach, such as logistic regression, only yielded prediction accuracy with an AUC of 0.837 and 0.822 in the validation dataset and the test dataset, respectively.

### 2.2. Risk Factors Identified by the DeepBiomarker Model with Significant Contributions

As previously mentioned, we used a perturbation-based estimation to determine the relative contribution (RC) of each feature in predicting AD + P. Out of the 65 features that demonstrated significant effects in our results, 23 were diagnoses; 36 were drugs, and 6 were lab tests. To enhance the reliability of our findings, we only included diagnoses that appeared in more than 10% of the entire population.

To provide a more comprehensive understanding of the significant features, we have provided three tables (Table 2, Table 3 and Table 4) that detail their specific effects. The RC values in these tables indicate the association of each feature with AD + P. An RC above 1 indicates that the feature contributes more to cases than controls, suggesting a hazardous effect, while an RC below 1 suggests a protective effect. These results shed light on the critical features that contribute to the development of AD + P and can be used to improve the accuracy of our predictive model. By identifying the specific risk factors associated with AD + P, we can design more targeted treatment and prevention strategies to improve patient outcomes.

Table 2 highlights several common comorbid diseases among AD patients, such as Diabetes, Esophageal Reflux, Atrial Fibrillation, Depression, and Primary Hypercoagulable State. These diseases have RC values greater than 1, indicating that they are associated with an increased risk of AD + P. Conversely, a few of the comorbidities, such as hypoxemia, pain in the joint/shoulder region, arthropathy, and osteoarthritis, have RC values less than 1, indicating that they are associated with a lower risk of AD + P.

Table 3 reveals several medications with RC values of less than 1 that are associated with reduced rates of onset of AD + P. These include hypertension medications, such as losartan and irbesartan, the hypotension drug midodrine, antidiabetic drug glipizide, anti-gastroesophageal reflux drugs pantoprazole, famotidine, sucralfate, and esomeprazole, laxatives docusate and lactulose, pain medications, such as aspirin, tramadol, and glucosamine-chondroitin, medications for chest pain, nitroglycerin and isosorbide mononitrate, cholesterol-lowering medication ezetimibe, asthma or chronic obstructive pulmonary disease drug budesonide-formoterol, antidepressants, duloxetine and cyclobenzaprine, vitamin D and fish oil, along with the antipsychotic quetiapine.

In contrast, Table 3 also identifies medications with RC values greater than 1 that are associated with a higher risk of developing AD + P. These include metoclopramide, an antiemetic and gut motility stimulator, allopurinol, uric acid reducer, warfarin, an anticoagulant, fluconazole, an antifungal drug, cholestyramine–aspartame, a cholesterol-lowering medication, and terazosin, an antihypertensive drug and urinary retention medication.

Interestingly, Table 4 reveals that all six lab tests with Q < 0.05, such as Chloride (Cl), Glucose, Urea Nitrogen, Anion Gap, Alkaline Phosphatase (ALP) Test, and Aspartate Aminotransferase (AST) Test, have RC values of less than 1 indicating that they are associated with a reduced risk of developing AD + P.

In general, this study built a cutting-edge deep-learning-based predictive model that can accurately predict the risk of an AD patient developing into an AD + P patient. In addition, this interpretable model allowed us to identify a set of risk factors and beneficial elements that may play important roles in the development of AD + P. These findings can further lead to novel therapeutics, mechanism explorations, and better treatment options.

## 3. Discussion

This study presents a significant stride forward in our understanding of risk factors and protective measures related to AD + P. By leveraging EMRs and deep learning models, we uncovered novel clinical features and potential pharmacological interventions that deserve further exploration. Notably, our work validates and extends previous research conducted at the University of Pittsburgh Alzheimer’s Disease Research Center (ADRC), reinforcing the protective role of vitamin D and uncovering the potential benefits of quetiapine, duloxetine, and memantine in reducing AD + P risk.

Our results have highlighted the significant beneficial effects of vitamin D, quetiapine, duloxetine, and memantine in reducing the risk of developing AD + P. In our previous study on research data collected by ADRC and network analysis, the use of vitamin D was associated with a lower risk of developing AD + P [36,37]. The strong beneficial effect of vitamin D in this observational study using EMR data further supports the potential beneficial effect of vitamin D in preventing AD + P. For quetiapine, though there are studies suggesting no significant difference in treating behavioral symptoms and cognitive decline, their effect in managing psychotic symptoms in AD + P remains unclear [38,39]. In an 8-week, double-blind, placebo-controlled trial, duloxetine exhibited a strong effect in improving cognition, depression, and some pain measures and was safe and well-tolerated in elderly patients [40]. As for memantine, mixed results have been reported for its potential effect in treating psychotic symptoms associated with dementia; some studies found that there is no significant beneficial effect between memantine and placebo in terms of their Positive and Negative Syndrome Scale (PANSS) score [41,42]. While some studies suggested that the use of memantine in addition to atypical antipsychotics can be beneficial in managing negative symptoms, several randomized, double-blind, placebo-controlled studies have reported that memantine alone showed a significant beneficial effect by decreasing the PANSS score [43,44,45].

In a deep learning model, the relationship between features can be complex and not entirely independent [46]. When estimating the effect of these features using a perturbation-based approach, the model’s ability to capture intricate patterns and dependencies between input features is taken into account. Perturbation-based methods involve making small changes to the input features and observing their impact on the model’s predictions [35]. By doing so, these methods can help disentangle the complex relationships and interactions among multiple features, providing insights into their individual and joint contributions to the model’s output.

Another point we should bear in mind when interpreting the results for the model is that compared to traditional statistical analysis (such as Logistic Regression or Survival Analysis), our deep learning model can take advantage of the features that showed high collinearity, such as the diagnosis of a disease and their treatments. Deep learning models are designed to capture complex relationships and interactions between input features [47]. During the training process, the model learns to recognize patterns and dependencies in the data, including correlations between different features. The model’s neurons can learn to capture interactions between features by combining them in various ways. For example, the model may learn to recognize that a specific medication is prescribed for a particular disease diagnosis. This information can be represented in the weights and biases of the neurons.

When we consider the combined effects of comorbidities and their treatments, we observe some interesting findings. For instance, joint disorders, such as arthropathy, osteoarthritis, and pain in the joint or shoulder region, are associated with inflammation. These tend to reduce the risk of developing AD + P. While this might seem surprising, it is plausible that patients with these conditions are more likely to take calcium/vitamin D supplements, which can target both peripheral and brain-specific inflammatory processes, ultimately reducing the risk of AD + P.

A key area of our investigation focused on glucose metabolism. Our findings highlight the pivotal role it plays in the development and severity of AD + P, a relationship that is intricate and warrants further exploration. Our study, for instance, identified diabetes as a risk factor for AD + P but found that the effects of treatments for diabetes on AD + P risk were mixed. Glipizide, an oral glucose-lowering drug, was associated with reduced AD + P risk, while the use of insulin was linked with an increased risk. These findings underline the potential for targeted therapies in this domain and invite a more comprehensive exploration of glucose metabolism’s role in the AD + P development [48]. 

Our research also shed light on the significant connection between cardiovascular disease and AD + P. Hypertension, a major risk for cerebrovascular disease, was not associated with AD + P risk, potentially due to the protective effects of hypertension medications, such as irbesartan and losartan. However, the complex pattern of cerebrovascular risk and its treatments, such as atrial fibrillation, aortic valve disorders, and their associated medications (clopidogrel and warfarin), call for an in-depth investigation into the underlying pharmacological mechanisms. A more complex pattern is highlighted by atrial fibrillation and aortic valve disorders, which both increase cerebrovascular risk and are associated with an increased risk of AD + P. However, the effects of their treatments differed. Clopidogrel was associated with reduced AD + P risk, suggesting that this therapy effectively mitigated the risks associated with their indications. In contrast, warfarin, which is also prescribed for these indications, was associated with an increased risk of AD + P. Examining the mechanisms and targets of medications that are associated with AD + P (Table 5) sheds further light on the reasons why these drugs with the same indications might have opposite effects on AD + P risk. Warfarin is an anticoagulant medication that inhibits the production of vitamin K-dependent clotting factors in the liver, whereas clopidogrel is an antiplatelet medication that inhibits the activation and aggregation of platelets. As warfarin and clopidogrel can penetrate the blood–brain barrier, they may exert central nervous system effects that are independent of their anticoagulant and antiplatelet functions. For example, it has been reported that clopidogrel can delay the closure of compromised blood–brain barriers by inhibiting the purinergic receptor P2RY12 [49]. More molecular mechanistic studies are needed to fully understand the underlying mechanisms for the beneficial effects of the medications identified in our study. Nonetheless, our findings provide important clues for the development of new medications that can target specific pathways and mechanisms involved in the development of AD + P. This has led us to key targets, such as adrenergic receptors and angiotensin II receptors, which are influenced by certain medications. The fact that cardiovascular-related drugs and conditions emerged as key factors in our analysis points to potential targets and mechanisms in this domain, such as adrenergic receptors and angiotensin II receptors [50]. The diverse effects of different antihypertensive medications suggest that the protective influence of certain drugs may not be solely attributed to their blood pressure-lowering effect, warranting further investigation. Intriguingly, not all antihypertensive medications demonstrated similar protective effects, as seen with Irbesartan and Losartan. This suggests that the beneficial effects of these drugs could be attributed to mechanisms beyond their blood-pressure-lowering capacity. The absence of similar effects in the case of diuretics and calcium channel blockers strengthens this assertion. Another salient point is the association between the impairment of mitochondrial functions and a range of neurological diseases, including AD, depression, anxiety, and psychiatric symptoms [51,52]. These findings underscore the need for further exploration to unravel the intricate mechanisms that contribute to AD + P’s development. This will aid in the identification of innovative therapeutic targets to effectively prevent or manage this disease subtype.

The significant association between the results of Aspartate Aminotransferase (AST) and Alkaline Phosphatase (ALP) tests and AD + P that we found in our study calls for an intriguing interpretation. Both AST and ALP are enzymes that, among other roles, are key indicators of liver and kidney function [53,54]. Their elevated levels often indicate some level of dysfunction or damage in these organs. The connection we’ve identified suggests that there could be an underlying link between kidney-related indicators and brain health, possibly through metabolic or vascular pathways. The metabolic waste products that the kidneys filter from the blood, for instance, might have a more intricate relationship with the brain’s health and function than previously understood [53,54]. If these waste products are not efficiently removed, they might indirectly impact brain health, potentially contributing to the development or exacerbation of AD + P. Likewise, kidney diseases are often associated with vascular problems, such as hypertension, which has been linked to cognitive decline and dementia [55]. This adds another layer of complexity to the relationship between kidney health and brain function. With these interpretations in mind, it is plausible that monitoring kidney health could be of great importance in the early detection, prevention, and management of AD + P. Health strategies could involve regular screenings for kidney function, especially in populations at risk of AD + P. Such measures could help to identify potential issues early, allowing for timely intervention. Furthermore, treatments aimed at improving kidney function or managing kidney diseases might also prove beneficial for AD + P patients.

The DeepBiomarker 1.5 model identified several important features that were strongly associated with AD + P, including inflammation-, glucose-metabolism-, cardiovascular-, and kidney-related biomarkers/mechanisms. Though the beneficial effect of glucose-lowering medications (glipizide, insulin) [56,57], cardiovascular medications (midodrine, irbesartan, losartan, clopidogrel, warfarin, terazosin, losartan) [58,59,60], antibiotics (cephalexin), topical corticosteroids (clobetasol), dietary supplements (glucosamine–chondroitin), and anti-inflammatory medications (triamcinolone acetonide, aspirin) [61] in AD have already been reported by multiple studies, there is no clear evidence supporting their correlation with AD + P. It is possible that these medications exert their protective effects against AD + P by treating comorbidities in AD. However, our findings also provide some mechanistic insight into the association between AD, AD + P, and these comorbidities. 

Our research has limitations that need to be taken into account when interpreting the results. First, because the patients had a limited number of medications, we could not assess the effects of medications with few to no users. Additionally, there may be inconsistencies in patients’ biochemical test results due to enrollment bias, and some laboratory tests may be underrepresented in our database, limiting the analysis’s ability to detect their effects. Furthermore, although we investigated the influence of biomarkers, comorbidities had a greater impact since the diagnosis considers the patients’ past status, while biomarkers only take into account their current status.

We would also like to point out that the population used in this study partially overlapped with the population in one of our previous studies that reported the beneficial effect of Vitamin D against AD + P [36]. We were unable to match and exclude the overlapping patients because of the deidentification process conducted by the data management team at the UPMC. However, with a total of 502 subjects included in the previous study, the overlapping sample size is too small to cause a significant impact.

Despite the potential limitations inherent to our observational study and the complexity of the identified dependencies among various health conditions and treatments, our findings pave the way for future research. They underline the need for a comprehensive approach that considers these interdependencies and advances toward more targeted preventive measures and therapeutic strategies for AD + P. Our study ultimately emphasizes the potential of big data and machine learning in this domain while underlining the need for an integrated approach that considers not only the primary disease symptoms but also co-existing conditions and their treatments. Our analysis has highlighted a novel angle in drug development for AD + P by revealing many drugs that have CNS penetration and impact varying protein targets present in the brain, and thus, may not impact AD + P through their effects on their original indications, but rather through other underlying mechanisms that are directly involved in the development of AD + P. These findings have important implications for drug development in AD + P and suggest that novel therapeutic targets should be explored to effectively prevent or treat this subtype. For example, looking at the overlap of the gene networks of these drugs’ targets within the CNS and that of AD + P may prove fruitful for the identification of new mechanisms for the prevention of AD + P [20,36]. Moving forward, our research emphasizes the necessity of a holistic approach to disease management. Future research should be directed toward disentangling the intricate web of dependencies among various health conditions and treatments. Ideally, the goal is to develop effective prevention and treatment strategies that account for these multifaceted interactions. To reach this goal, it is crucial to expand upon our findings with more detailed mechanistic studies and randomized clinical trials. In the grand scheme of the field, this research further underscores the immense potential of big data and machine learning in advancing our understanding of such complex diseases as AD + P. At the same time, it also emphasizes the critical role of a comprehensive and integrative approach, considering not only primary disease symptoms but also co-existing conditions and their treatments.

## 4. Materials and Methods

The application of deep learning models to predict clinical outcomes using electronic medical records (EMR) data has gained significant attention recently [62,63]. EMR data, which typically represents a patient’s history as a sequence of visits with multiple events per visit, is well-suited for such sequence models as RNNs [64,65]. Recent studies indicated that simple-gated RNN models, such as Gated Recurrent Units (GRUs) and Long Short-Term Memory (LSTMs), when finely tuned using Bayesian Optimization, often deliver competitive outcomes [33]. Due to the limited sample size, we did not use Transformer-based models, which require a large amount of data for training.

### 4.1. Data Source

We examined the data from January 2004 to October 2019 from the Neptune system at the UPMC, which manages the use of patient EMRs from the UPMC health system for research purposes (rio.pitt.edu/services, accessed on 2 May 2023) [66]. The database includes demographic information, diagnoses, encounters, medication prescriptions, prescription fill history, and laboratory tests. AD patients and psychosis patients were identified using a series of diagnosis terms in the EMR systems (Supplementary Lists S1 and S2). In addition, to avoid the possible misdiagnosis of psychosis by short-term delirium symptoms, a psychosis diagnosis that co-occurred with a delirium diagnosis (Supplementary List S3) was excluded.

### 4.2. Inclusion/Exclusion Criteria and Data Preparation

For each AD patient, we would like to predict whether the patient will have psychosis within the next 3 months, given the history of EMRs. The inclusion criteria for cases and controls are described as follows. To be included in this study, an AD patient had to have at least a one-year EMR record prior to the first AD diagnosis, and the patient had no previous history of psychosis diagnosis or at least a one-year washout period before AD. This is to make sure that (1) we have collected enough comorbidity information for this patient and (2) the psychosis onset was new to this patient after the AD diagnosis. At any encounter, an AD patient who had a record of psychosis within the following 3 months is defined as a case, while no record of psychosis within the following 3 months is defined as a control. For a patient with multiple encounters satisfying the criteria of control, only the last encounter was included to mimic the latest status of these patients. We also require no records of psychosis during this period to the index date to make sure that this is a new onset of AD + P. Moreover, we used data augmentation to increase the number of cases (see below). The date of this encounter will be the index date. We used the medication, diagnosis, and lab tests 1 year preceding the index date as the input. For lab tests, we only included those abnormal ones in our modeling by searching those RESULT_FLAG labeled as “ABNORMAL”, “HIGH”, or “LOW”. We also excluded those lab tests with low frequency and kept the 89 top frequently tested ones. The diagnosis was coded in ICD9 before the year 2015 and ICD10 after the year 2015. As such, we used a lookup table from https://www.cms.gov/Medicare/Coding/ICD10/2018-ICD-10-CM-and-GEMs (accessed on 8 December 2022) to convert ICD9 to ICD10 codes. The first three characters of the ICD10, which designate the category of the diagnosis, were extracted, yielding 1614 diagnosis groups. Medication names were converted to DrugBank IDs by name matching, and 1407 unique DrugBank IDs were mapped. Finally, for each encounter, the associated medications, diagnosis, and abnormal lab test results were packed into a sequence with the indices of DrugBank IDs, categories of the diagnosis, and lab test IDs, respectively. 

### 4.3. Data Augementation

**Figure 1:**
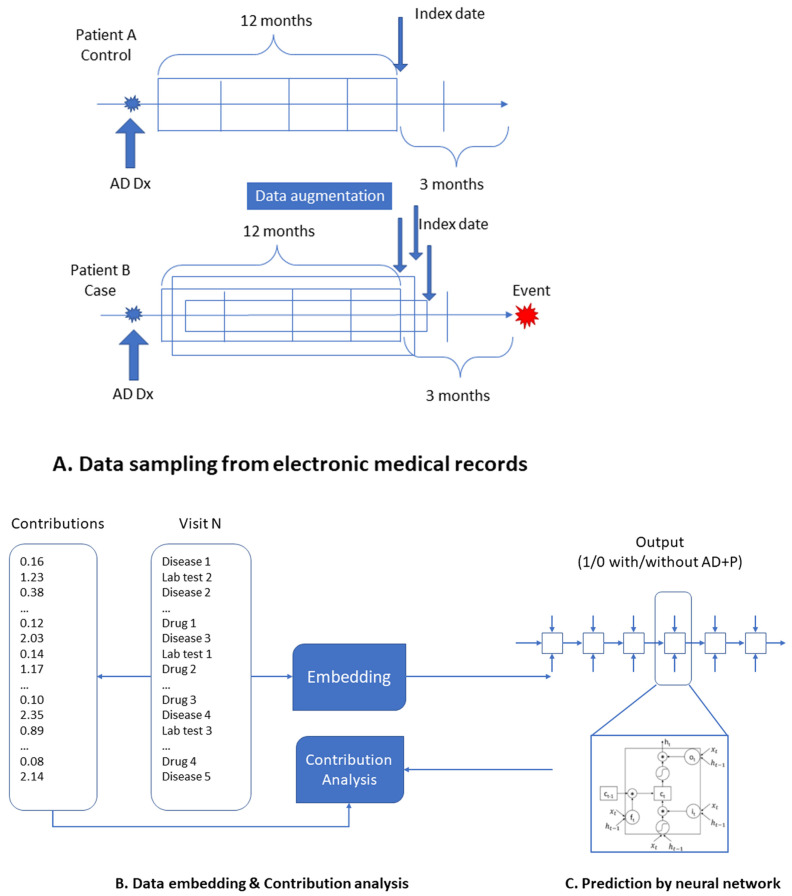
Overview of DeepBiomarker. (**A**) Data sampling process from EMRs to create case and control cohorts; data augmentation was applied to oversample AD + P patients to create balanced datasets. (**B**) Data embedding is a process of representing higher dimensional data in a lower-dimensional space while preserving the relevant properties of the original data. (**C**) Prediction by neural network with LSTM as the basic prediction unit. Perturbation-based contribution analysis was used to identify important features.

Data augmentation is a technology used to increase the data size and reduce overfitting. At any encounter, the chance of having psychosis within the next 3 months was much lower than that of having no psychosis, even within these AD patients with high risk. We included all encounters nearby the psychosis, which satisfied the inclusion criteria for positive cases while under-sampling the encounters which satisfied the inclusion criteria for controls (Figure 1). The purpose of data augmentation is to enhance the influence of factors nearby the events while reducing the effects of factors far from the events. 

The dataset was split with a ratio of 8:1:1, and 8 of 10 subsets were used as the training dataset, while 1 of 10 subsets was used as the validation dataset to find the optimal parameters, and 1 subset was used as the test set to evaluate the generalization of our model. 

### 4.4. Model Construction and Assessment

We adopted the Pytorch_EHR framework established by ZhiGroup, where Deep learning models with Vanilla RNN, GRU, LSTM, Bidirectional RNN, Bidirectional GRU, Bidirectional LSTM, Dilated RNN, Dilated GRU, Dilated LSTM, QRNN, and T-LSTM were used to analyze and predict clinical outcomes [33]. We further modified the framework, as highlighted in Figure 1, by (A) data augmenting to improve the model performance, (B) including individual lab tests and medications along with the diagnosis groups as the input so that we could assess the effects of each lab tests and medications, and (C) integrating contribution analysis [35] module for the importance estimation of key factors (see below for more details). The structure we used here was the LSTM model, which stores previous illness history, infers current illness states, and predicts future medical outcomes. The memory cell is gated to moderate the information flow to or from the cell. LSTMs have been adapted to many applications, such as machine translation, handwriting recognition, and speech recognition. In this study, the following parameters are used: embed dimension: 128; hidden size: 128; dropout rate: 0.2; the number of layers: 2; input size: 30,000; patience: 3. The calculations were repeated ten times for each deep learning algorithm to estimate the standard deviations of the accuracy. 

To further investigate the importance of those factors on the prediction of psychosis, we calculated the relative contribution (*RC*) of each feature on the psychosis [35]. The *RC* of a feature was calculated as the average contribution of the feature to events divided by the average contributions of this feature to no events. The contributions were estimated by a perturbation-based approach. Such an approach has been used in a recent study on the important features of the heart failure incidence prediction [67]. The equation is shown as follows, where *FC* represents the feature contribution:

(1) $$RC=\frac{mean\;FC\;in\;patients\;with\;event}{mean\;FC\;in\;patients\;without\;event}$$

*FC* value was the total value of the feature within the same patient if the feature appeared more than once in that patient. The natural logarithm form variance for *RC* was calculated as follows:

(2) $$Variance\left(\ln(RC\right))=\frac{{\left(\frac{sd\;of\;FC\;of\;patients\;with\;event}{mean\;of\;FC\;of\;patients\;with\;event}\right)}^{2}}{number\;of\;patients\;with\;event}+\frac{{\left(\frac{sd\;of\;FC\;of\;patients\;without\;event}{mean\;of\;FC\;of\;patients\;without\;event}\right)}^{2}}{number\;of\;patients\;without\;event}$$

Thus, the 95% confidence interval (CI) of *RC* was given by

(3) $$95\%\mathrm{CI}=e^{(\ln\left(RC\right))\pm 1.96\sqrt{Variance\left(\ln\left(RC\right)\right)}}$$

The *p*-value was under the assumption of z distribution [68]. Bonferroni correction [69] was used to reduce the type I error caused by multiple comparisons.

In this version of DeepBiomarker (V1.5), we refined our RC calculation by normalizing the FC value and scaling the RC values for all our features. The improved FC formula is the ratio of the summary of the contribution of a feature and the summary of the contribution of all the features. Next, we performed scaling, where the RC for an AD diagnosis was scaled to 1, and the factor generated was multiplied to get the real RC value for each of the other features. The rationale of normalization is to consider the different occurrences of a feature in patients because of differences in the number of encounters and consider the influence of other features. The rationale for scaling the RC of AD to 1 is that all the patients had AD, and AD will have similar effects on the risk of psychosis.

Model performance was evaluated by the area under the ROC curve (AUROC).

## 5. Conclusions

In this study, we presented a cutting-edge deep learning model, DeepBiomarker, that is capable of accurately predicting the onset the AD + P and identifying risk factors and potential treatment options for AD + P. The results generated by this study not only provided a powerful tool for clinical care but also provided insights for mechanism studies related to AD + P and can further facilitate the development of effective treatment options for AD + P.

Our research emphasizes the remarkable potential of big data and deep learning in unraveling the multifaceted influences on the development of AD+P. Through our work, we spotlight the intricate interplay of comorbid conditions and their treatments in AD+P pathogenesis, highlighting glucose metabolism, cardiovascular factors, and kidney/liver functions as key areas of interest. This provides a valuable springboard for targeted therapies that extend beyond conventional symptom management. However, the complexity of these interdependencies necessitates refined methodologies for accurate interpretation, emphasizing a need for comprehensive, holistic approaches in both research and clinical practice. Future research should aim to disentangle these relationships further, fostering the development of innovative preventive measures and therapeutic strategies for AD + P. This study reaffirms the pivotal role of translational research, bridging the gap between theory and clinical application as we continue our pursuit to alleviate the burden of Alzheimer’s disease.

## Figures and Tables

**Table 1:** Performance of three AD + P predicting models.

	Validation AUC	Test AUC	Validation AUC Std.	Test AUC Std.
T-LSTM	0.921	0.903	0.006	0.005
RETAIN	0.935	0.907	0.004	0.002
LR	0.837	0.822	0.009	0.012

AUC: Area under the receiver operating characteristic curve. Std: Standard deviation. T-LSTM: Temporal information enhancing long short-term memory. RETAIN: Reverse time attention model. LR: Logistic regression.

**Table 2:** Relative contributions of diagnosis features that showed significant association (Q < 0.05) with AD + P, listed in order of increasing RC value.

Feature	RC	CI95up	CI95down	Q Value *
Hypoxemia	0.718	0.85	0.606	0.002
Arthropathy, unspecified, site unspecified ^1^	0.747	0.831	0.672	<0.001
Pain in joint, shoulder region	0.764	0.898	0.651	0.01
Activities involving walking, marching, and hiking	0.782	0.876	0.699	0.001
Acute kidney failure, unspecified ^1^	0.86	0.945	0.783	0.014
Unspecified osteoarthritis, unspecified site ^1^	0.877	0.944	0.814	0.006
Esophageal reflux	1.112	1.174	1.054	0.002
Depressive disorder, not elsewhere classified	1.117	1.195	1.045	0.01
Hypothyroidism, unspecified ^1^	1.136	1.234	1.045	0.02
Disorientation, unspecified ^1^	1.148	1.268	1.039	0.039
Atherosclerotic heart disease of native coronary artery without angina pectoris	1.154	1.228	1.085	<0.001
Abnormality of gait	1.171	1.292	1.061	0.014
Type 2 diabetes mellitus without complications	1.191	1.261	1.125	<0.001
Obstructive sleep apnea	1.207	1.354	1.076	0.012
Central pain syndrome	1.221	1.397	1.067	0.025
Diabetes mellitus without mention of complication, type II or unspecified type, not stated as uncontrolled ^1^	1.234	1.328	1.147	<0.001
Aortic valve disorders	1.274	1.506	1.077	0.03
Atrial fibrillation	1.358	1.477	1.249	<0.001
Dependence on renal dialysis	1.361	1.602	1.156	0.003
Hypocalcemia	1.417	1.689	1.189	0.002
Long term (current) use of insulin	1.582	1.722	1.454	<0.001
Primary hypercoagulable state	1.582	1.722	1.454	<0.001
Acute venous embolism and thrombosis of unspecified deep vessels of lower extremity	1.687	2.325	1.223	0.012

* FDR-adjusted *p*-value. ^1^ The term “unspecified” is used in ICD9 and ICD10 codes when the available medical record information is not sufficient to assign a more specific code. RC: relative contribution; CI: confidential interval.

**Table 3:** Relative contributions of medication features that showed significant association (Q < 0.05) with AD + P, listed in order of increasing RC value.

Feature	Indication/Drug Class	RC	CI95up	CI95down	Q Value *
Glucosamine–Chondroitin	Osteoarthritis, reduce joint pain and inflammation	0.359	0.7	0.184	0.02
Dextromethorphan–Guaifenesin	Cough suppressant	0.439	0.757	0.255	0.022
Fish Oil	Dietary supplement	0.456	0.73	0.285	0.01
Sucralfate	Gastrointestinal ulcers	0.472	0.716	0.311	0.005
Midodrine	Alpha-adrenergic agonist for hypotension	0.477	0.65	0.35	<0.001
Irbesartan	Angiotensin receptor blocker for hypertension	0.509	0.769	0.338	0.012
Esomeprazole Magnesium	Proton pump inhibitor	0.538	0.698	0.414	<0.001
Cyclobenzaprine	Skeletal muscle relaxant	0.572	0.734	0.445	<0.001
Budesonide–Formoterol	Corticosteroid/beta2-adrenergic receptor agonist	0.578	0.763	0.437	0.002
Lactulose	Constipation and portal systemic encephalopathy	0.599	0.835	0.429	0.019
Duloxetine	Antidepressant	0.606	0.757	0.485	<0.001
Ezetimibe	Hyperlipidemia	0.624	0.854	0.457	0.022
Magnesium Hydroxide	Laxative and antacid	0.66	0.823	0.529	0.003
Famotidine	Histamine H2 receptor antagonist	0.672	0.823	0.548	0.002
Nitroglycerin	Nitrate vasodilator	0.679	0.84	0.549	0.005
Alprazolam	Anxiety disorders and panic disorders	0.692	0.857	0.56	0.008
Isosorbide Mononitrate	Prevent and treat angina in coronary artery disease	0.719	0.89	0.582	0.018
Quetiapine	Antipsychotics	0.726	0.875	0.603	0.008
Glipizide	Type 2 diabetes	0.732	0.923	0.581	0.046
Memantine	Alzheimer’s disease	0.749	0.83	0.676	<0.001
Triamcinolone Acetonide	Corticosteroid	0.751	0.923	0.612	0.038
Losartan	Angiotensin receptor blocker for hypertension	0.766	0.869	0.676	0.001
Clopidogrel	Antiplatelet	0.777	0.918	0.658	0.022
Docusate Sodium	Stool softener	0.78	0.907	0.671	0.011
Calcium Carbonate-Vitamin D3	Calcium supplement/osteoporosis	0.781	0.902	0.676	0.008
Cephalexin	Antibiotics	0.793	0.917	0.686	0.014
Tramadol	Opioid agonist and serotonin/norepinephrine reuptake inhibitor	0.795	0.937	0.674	0.037
Aspirin	Antiplatelet/Non-steroidal anti-inflammatory drugs	0.808	0.904	0.721	0.003
Pantoprazole	Proton pump inhibitor	0.835	0.95	0.734	0.036
Warfarin	Anticoagulated/vitamin K antagonist	1.289	1.478	1.124	0.004
Fluconazole	Antifungal medication	1.58	2.175	1.147	0.032
Allopurinol	Xanthine oxidase inhibitor/reduce uric acid concentrations	1.639	2.157	1.245	0.005
Cholestyramine–Aspartame	Lower cholesterol levels	1.642	2.233	1.207	0.013
Terazosin	Alpha-1 adrenergic antagonist/hypertension	1.842	2.633	1.288	0.009
Metoclopramide	Antiemetic agent and dopamine D2 antagonist	1.879	2.697	1.309	0.007
Clobetasol	High potency corticosteroid topical medication	2.054	3.043	1.387	0.004

* FDR-adjusted *p*-value. RC: relative contribution; CI: confidential interval.

**Table 4:** Relative contributions of lab test features that showed significant association (Q < 0.05) with AD + P.

Feature	RC	CI95up	CI95down	Q Value *
Aspartate Aminotransferase (AST) Test	0.704	0.864	0.574	0.008
Alkaline Phosphatase (ALP) Test	0.826	0.924	0.739	0.009
Urea Nitrogen	0.84	0.909	0.776	0.001
Anion Gap	0.863	0.944	0.789	0.012
Glucose	0.871	0.941	0.806	0.006
Chloride (Cl)	0.886	0.952	0.825	0.01

* FDR-adjusted *p*-value. RC: relative contribution; CI: confidential interval.

**Table 5:** Mechanism actions, targets, and blood–brain barrier (BBB) penetration ability for the medications identified associated with the development of AD + P.

Drugs	Mechanism of Action	Targets *	Ability to Penetrate Blood–Brain Barrier	Predicted Effects against AD + P
Glucosamine–Chondroitin	Maintain healthy cartilage by providing the building blocks for its synthesis and supporting its repair	UDP-glucose 2-epimerase/ManNAc kinase (GNE) gene	No	Beneficial
Dextromethorphan–Guaifenesin	Cough suppressant that works by acting on the cough center in the brain/thinning and loosening mucus in the airways	GRIN1 GRIN2A GRIN2B SIGMAR1 HTR3A HTR3B	Yes	Beneficial
Fish Oil	Incorporating into cell membranes and modulating the production of eicosanoids		Yes	Beneficial
Sucralfate	Forming a protective barrier over the ulcer or damaged area, which helps to prevent further damage and promote healing		No	Beneficial
Midodrine	Selective alpha-1 adrenergic agonist, which increases peripheral vascular resistance and blood pressure	ADRA1A	No	Beneficial
Irbesartan	Selectively blocking the angiotensin II receptor type 1 (AT1) in the renin–angiotensin–aldosterone system, which leads to vasodilation and a decrease in blood pressure	AT1 AGTR1	No	Beneficial
Esomeprazole Magnesium	Inhibiting the proton pump (H+/K+ ATPase) in the stomach	the proton pump (H+/K+ ATPase)	Yes	Beneficial
Cyclobenzaprine	A centrally-acting muscle relaxant, which reduces muscle tone and spasm by blocking the activity of alpha motor neurons in the spinal cord	alpha motor neurons in the spinal cord	Yes	Beneficial
Budesonide–Formoterol	Binding to glucocorticoid receptors in the lungs, leading to the suppression of inflammation and immune responses	Glucocorticoid receptors Beta-2 adrenergic receptors	Yes	Beneficial
Lactulose				Beneficial
Duloxetine	Inhibition of the reuptake of two neurotransmitters in the brain, serotonin and norepinephrine	SLC6A2 SLC6A4	Yes	Beneficial
Ezetimibe	Increasing the osmotic pressure in the colon, which draws water into the colon and softens the stool	NPC1L1 SOAT1	No	Beneficial
Magnesium Oxide	Providing magnesium ions to the body, which are essential for many biological processes		Yes	Beneficial
Famotidine	Inhibiting the activity of histamine h2 receptors in the stomach	histamine H2 receptor	Yes	Beneficial
Nitroglycerin	A potent vasodilator by releasing nitric oxide in the smooth muscle of blood vessels, leading to relaxation of vascular smooth muscle and vasodilation	NPR1	Yes	Beneficial
Alprazolam	Enhancing the activity of gamma-aminobutyric acid (GABA) in the brain	GABA-A receptor benzodiazepine receptor	Yes	Beneficial
Isosorbide Mononitrate	It acts as a vasodilator by releasing nitric oxide in the smooth muscle of blood vessels, leading to relaxation of vascular smooth muscle and vasodilation	NPR1	No	Beneficial
Quetiapine	Antagonist of several neurotransmitter receptors in the brain, including dopamine, serotonin, and histamine receptors	DRD2 HTR1A HTR2A HRH1	Yes	Beneficial
Glipizide	Stimulating the release of insulin from the beta cells of the pancreas.	ATP-sensitive potassium channels in pancreatic beta cells SUR1	No	Beneficial
Memantine	Blocking of the activity of the NMDA (n-methyl-d-aspartate) subtype of glutamate receptors in the brain.	NMDA subtype of glutamate receptors	Yes	Beneficial
Triamcinolone Acetonide	A synthetic glucocorticoid, which reduces inflammation and swelling by inhibiting the production and release of inflammatory mediators	Inflammatory mediators and their signaling pathways. NR3C1	No	Beneficial
Losartan	Angiotensin II receptor antagonist, blocking the binding of angiotensin II to specific receptors in the body, which inhibits its vasoconstrictive and pro-inflammatory effects	angiotensin II receptor	Yes	Beneficial
Clopidogrel	Irreversibly inhibits the P2Y12 receptor, which is found on the surface of platelets. Reduces the activation and aggregation of platelets.	P2Y12 receptor	Yes	Beneficial
Docusate Sodium	Increasing the amount of water and fat in the stool		No	Beneficial
Vitamin D	Binding to vitamin d receptors (VDR) in cells, leading to changes in gene expression and protein synthesis	Vitamin D receptor (VDR)	Yes	Beneficial
Cephalexin	Inhibiting bacterial cell wall synthesis by binding to penicillin-binding proteins (PBPS)	bacterial PBPs	No	Beneficial
Tramadol	An opioid agonist, which means it binds to and activates opioid receptors in the brain, inhibits the reuptake of serotonin and norepinephrine, which are neurotransmitters involved in pain processing, further enhancing its analgesic effect	OPRM1 SLC6A2 SLC6A4 SCN2A NMDA receptors ADORA1	Yes	Beneficial
Aspirin	Irreversibly inhibit the cyclooxygenase (COX) enzyme	PTGS1 PTGS2 AKR1C1 EDNRA TP53 HSPA5 RPS6KA3 NFKBIA	Yes	Beneficial
Pantoprazole	Irreversibly blocking the H+/K+-atpase enzyme in the parietal cells of the stomach	ATP4A ATP4B	No	Beneficial
Warfarin	Inhibiting the synthesis of vitamin K-dependent clotting factors in the liver, specifically factors II, VII, IX, and X	VKORC1 NR1I2	Yes	Hazardous
Fluconazole	Inhibiting fungal cytochrome P450-dependent enzymes	fungal cytochrome P450-dependent enzymes	Yes	Hazardous
Allopurinol	Inhibiting the xanthine oxidase enzyme, which is involved in the metabolism of purines	xanthine oxidase enzyme	Yes	Hazardous
Cholestyramine–Aspartame	Binding to bile acids in the intestine and preventing their reabsorption	bile acids	No	Hazardous
Terazosin	Blocking the alpha-1 adrenergic receptors in smooth muscle tissue, including the prostate and blood vessels	ADRA1A ADRA1B ADRA1D	No	Hazardous
Metoclopramide	Blocking dopamine receptors and stimulating 5-HT4 serotonin receptors in the gastrointestinal tract	DRD1 DRD2 DRD3 DRD4 DRD5 HTR4	Yes	Hazardous
Clobetasol	Binding to and activating glucocorticoid receptors in skin cells	NR3C1	No	Hazardous

* Some of the medications have unclear mechanism of action/targets.

## Data Availability

Data are available upon request from the authors.

## Supplementary Materials

The following supporting information can be downloaded at: https://www.mdpi.com/article/10.3390/ph16070911/s1. Supplementary material contains: Supplementary List S1: diagnosis terms used to identify AD patients. Supplementary List S2: diagnosis terms used to identify psychosis patients. Supplementary List S3: diagnosis terms used to identify delirium patients.

## References

[1] Alzheimer’s Association (2016). 2016 Alzheimer’s disease facts and figures. Alzheimers Dement..

[2] Thorgrimsen L., Selwood A., Spector A., Royan L., de Madariaga Lopez M., Woods R., Orrell M. (2003). Whose quality of life is it anyway?: The validity and reliability of the Quality of Life-Alzheimer’s Disease (QoL-AD) scale. Alzheimer Dis. Assoc. Disord..

[3] Wimo A., Prince M. (2010). World Alzheimer Report. The Global Economic Impact of Dementia.

[4] Murray P.S., Kumar S., Demichele-Sweet M.A., Sweet R.A. (2014). Psychosis in Alzheimer’s disease. Biol. Psychiatry.

[5] Ropacki S.A., Jeste D.V. (2005). Epidemiology of and risk factors for psychosis of Alzheimer’s disease: A review of 55 studies published from 1990 to 2003. Am. J. Psychiatry.

[6] Battaglia S., Nazzi C., Thayer J.F. (2023). Fear-induced bradycardia in mental disorders: Foundations, current advances, future perspectives. Neurosci. Biobehav. Rev..

[7] Lanctôt K.L., Amatniek J., Ancoli-Israel S., Arnold S.E., Ballard C., Cohen-Mansfield J., Ismail Z., Lyketsos C., Miller D.S., Musiek E. (2017). Neuropsychiatric signs and symptoms of Alzheimer’s disease: New treatment paradigms. Alzheimers Dement..

[8] Lyketsos C.G., Carrillo M.C., Ryan J.M., Khachaturian A.S., Trzepacz P., Amatniek J., Cedarbaum J., Brashear R., Miller D.S. (2011). Neuropsychiatric symptoms in Alzheimer’s disease. Alzheimers Dement..

[9] Battaglia S., Di Fazio C., Vicario C.M., Avenanti A. (2023). Neuropharmacological Modulation of N-methyl-D-aspartate, Noradrenaline and Endocannabinoid Receptors in Fear Extinction Learning: Synaptic Transmission and Plasticity. Int. J. Mol. Sci..

[10] Sweet R.A., Bennett D.A., Graff-Radford N.R., Mayeux R. (2010). Assessment and familial aggregation of psychosis in Alzheimer’s disease from the National Institute on Aging Late Onset Alzheimer’s Disease Family Study. Brain J. Neurol..

[11] Krivinko J.M., Erickson S.L., Ding Y., Sun Z., Penzes P., MacDonald M.L., Yates N.A., Ikonomovic M.D., Lopez O.L., Sweet R.A. (2018). Synaptic Proteome Compensation and Resilience to Psychosis in Alzheimer’s Disease. Am. J. Psychiatry.

[12] DeChellis-Marks M.R., Wei Y., Ding Y., Wolfe C.M., Krivinko J.M., MacDonald M.L., Lopez O.L., Sweet R.A., Kofler J. (2022). Psychosis in Alzheimer’s Disease Is Associated With Increased Excitatory Neuron Vulnerability and Post-transcriptional Mechanisms Altering Synaptic Protein Levels. Front. Neurol..

[13] Krivinko J., DeChellis-Marks M., Zeng L., Fan P., Lopez O., Ding Y., Wang L., Kofler J., MacDonald M., Sweet R. (2023). Targeting the Post-Synaptic Proteome in Alzheimer Disease with Psychosis. Commun. Biol..

[14] Madhusoodanan S., Shah P. (2008). Management of psychosis in patients with Alzheimer’s disease: Focus on aripiprazole. Clin. Interv. Aging.

[15] Alexopoulos G.S., Streim J., Carpenter D., Docherty J.P. (2004). Expert consensus guidelines for using antipsychotic agents in older patients. J. Clin. Psychiatry.

[16] Burke A.D., Burke W.J. (2018). Antipsychotics FOR patients WITH dementia: The road less traveled: Second-generation agents have an important but limited role in treating behavioral and psychological symptoms. Curr. Psychiatry.

[17] Dorsey E.R., Rabbani A., Gallagher S.A., Conti R.M., Alexander G.C. (2010). Impact of FDA black box advisory on antipsychotic medication use. Arch. Intern. Med..

[18] Emanuel J.E., Lopez O.L., Houck P.R., Becker J.T., Weamer E.A., Demichele-Sweet M.A., Kuller L., Sweet R.A. (2011). Trajectory of cognitive decline as a predictor of psychosis in early Alzheimer disease in the cardiovascular health study. Am. J. Geriatr. Psychiatry Off. J. Am. Assoc. Geriatr. Psychiatry.

[19] Tampi R.R., Tampi D.J., Balachandran S., Srinivasan S. (2016). Antipsychotic use in dementia: A systematic review of benefits and risks from meta-analyses. Ther. Adv. Chronic Dis..

[20] Fan P., Kofler J., Ding Y., Marks M., Sweet R.A., Wang L. (2022). Efficacy difference of antipsychotics in Alzheimer’s disease and schizophrenia: Explained with network efficiency and pathway analysis methods. Brief. Bioinform..

[21] DeMichele-Sweet M.A.A., Klei L., Creese B., Harwood J.C., Weamer E.A., McClain L., Sims R., Hernandez I., Moreno-Grau S., Tarraga L. (2021). Genome-wide association identifies the first risk loci for psychosis in Alzheimer disease. Mol. Psychiatry.

[22] Bote-Curiel L., Muñoz-Romero S., Gerrero-Curieses A., Rojo-Álvarez J.L. (2019). Deep Learning and Big Data in Healthcare: A Double Review for Critical Beginners. Appl. Sci..

[23] Salehinejad H., Sankar S., Barfett J., Colak E., Valaee S.J. (2017). Recent advances in recurrent neural networks. arXiv.

[24] Vaswani A., Shazeer N., Parmar N., Uszkoreit J., Jones L., Gomez A.N., Kaiser Ł., Polosukhin I. (2017). Attention is all you need. Adv. Neural Inf. Process. Syst..

[25] Supriya M., Deepa A.J. (2020). Machine learning approach on healthcare big data: A review. Big Data Inf. Anal..

[26] Pham T., Tran T., Phung D., Venkatesh S. Deepcare: A deep dynamic memory model for predictive medicine. Proceedings of the Pacific-Asia Conference on Knowledge Discovery and Data Mining.

[27] Dernoncourt F., Lee J.Y., Uzuner O., Szolovits P. (2017). De-identification of patient notes with recurrent neural networks. J. Am. Med. Inform. Assoc..

[28] Che Z., Purushotham S., Khemani R., Liu Y. (2015). Distilling knowledge from deep networks with applications to healthcare domain. arXiv.

[29] Cheng Y., Wang F., Zhang P., Hu J. Risk prediction with electronic health records: A deep learning approach. Proceedings of the SIAM International Conference on Data Mining.

[30] Choi Y., Chiu C.Y., Sontag D. (2016). Learning Low-Dimensional Representations of Medical Concepts. AMIA Summits Transl. Sci. Proc..

[31] Shickel B., Tighe P.J., Bihorac A., Rashidi P. (2018). Deep EHR: A Survey of Recent Advances in Deep Learning Techniques for Electronic Health Record (EHR) Analysis. IEEE J. Biomed. Health Inform..

[32] Wiemken T.L., Kelley R.R. (2020). Machine Learning in Epidemiology and Health Outcomes Research. Annu. Rev. Public. Health.

[33] Rasmy L., Zhu J., Li Z., Hao X., Tran H.T., Zhou Y., Tiryaki F., Xiang Y., Xu H., Zhi D. (2021). Simple Recurrent Neural Networks is all we need for clinical events predictions using EHR data. arXiv.

[34] Miranda O., Fan P., Qi X., Yu Z., Ying J., Wang H., Brent D.A., Silverstein J.C., Chen Y., Wang L. (2022). DeepBiomarker: Identifying Important Lab Tests from Electronic Medical Records for the Prediction of Suicide-Related Events among PTSD Patients. J. Pers. Med..

[35] Guan C., Wang X., Zhang Q., Chen R., He D., Xie X. Towards a deep and unified understanding of deep neural models in nlp. Proceedings of the International Conference on Machine Learning.

[36] Wang L., Ying J., Fan P., Weamer E.A., DeMichele-Sweet M.A.A., Lopez O.L., Kofler J.K., Sweet R.A. (2019). Effects of Vitamin D Use on Outcomes of Psychotic Symptoms in Alzheimer Disease Patients. Am. J. Geriatr. Psychiatry Off. J. Am. Assoc. Geriatr. Psychiatry.

[37] Fan P., Qi X., Sweet R.A., Wang L. (2020). Network systems pharmacology-based mechanism study on the beneficial effects of vitamin d against psychosis in Alzheimer’s disease. Sci. Rep..

[38] Calsolaro V., Antognoli R., Okoye C., Monzani F. (2019). The Use of Antipsychotic Drugs for Treating Behavioral Symptoms in Alzheimer’s Disease. Front. Pharmacol..

[39] Vigen C.L., Mack W.J., Keefe R.S., Sano M., Sultzer D.L., Stroup T.S., Dagerman K.S., Hsiao J.K., Lebowitz B.D., Lyketsos C.G. (2011). Cognitive effects of atypical antipsychotic medications in patients with Alzheimer’s disease: Outcomes from CATIE-AD. Am. J. Psychiatry.

[40] Raskin J., Wiltse C.G., Siegal A., Sheikh J., Xu J., Dinkel J.J., Rotz B.T., Mohs R.C. (2007). Efficacy of duloxetine on cognition, depression, and pain in elderly patients with major depressive disorder: An 8-week, double-blind, placebo-controlled trial. Am. J. Psychiatry.

[41] Lee J.G., Lee S.W., Lee B.J., Park S.W., Kim G.M., Kim Y.H. (2012). Adjunctive memantine therapy for cognitive impairment in chronic schizophrenia: A placebo-controlled pilot study. Psychiatry Investig..

[42] Lieberman J.A., Papadakis K., Csernansky J., Litman R., Volavka J., Jia X.D., Gage A., Group M.-M.-S. (2009). A randomized, placebo-controlled study of memantine as adjunctive treatment in patients with schizophrenia. Neuropsychopharmacol. Off. Publ. Am. Coll. Neuropsychopharmacol..

[43] Veerman S.R., Schulte P.F., Smith J.D., de Haan L. (2016). Memantine augmentation in clozapine-refractory schizophrenia: A randomized, double-blind, placebo-controlled crossover study. Psychol. Med..

[44] Krivoy A., Weizman A., Laor L., Hellinger N., Zemishlany Z., Fischel T. (2008). Addition of memantine to antipsychotic treatment in schizophrenia inpatients with residual symptoms: A preliminary study. Eur. Neuropsychopharmacol. J. Eur. Coll. Neuropsychopharmacol..

[45] Omranifard V., Rajabi F., Mohammadian-Sichani M., Maracy M. (2015). The effect of add-on memantine on global function and quality of life in schizophrenia: A randomized, double-blind, controlled, clinical trial. Adv. Biomed. Res..

[46] Kotsiantis S.B., Zaharakis I., Pintelas P.J. (2007). Supervised machine learning: A review of classification techniques. Emerg. Artif. Intell. Appl. Comput. Eng..

[47] LeCun Y., Bengio Y., Hinton G. (2015). Deep learning. Nature.

[48] Kinney J.W., Bemiller S.M., Murtishaw A.S., Leisgang A.M., Salazar A.M., Lamb B.T. (2018). Inflammation as a central mechanism in Alzheimer’s disease. Alzheimers Dement..

[49] Lou N., Takano T., Pei Y., Xavier A.L., Goldman S.A., Nedergaard M. (2016). Purinergic receptor P2RY12-dependent microglial closure of the injured blood-brain barrier. Proc. Natl. Acad. Sci. USA.

[50] Huang Y. (2010). Mechanisms linking apolipoprotein E isoforms with cardiovascular and neurological diseases. Curr. Opin. Lipidol..

[51] Tanaka M., Szabó Á., Vécsei L. (2023). Preclinical modeling in depression and anxiety: Current challenges and future research directions. Adv. Clin. Exp. Med..

[52] Tanaka M., Szabó Á., Spekker E., Polyák H., Tóth F., Vécsei L. (2022). Mitochondrial Impairment: A Common Motif in Neuropsychiatric Presentation? The Link to the Tryptophan—Kynurenine Metabolic System. Cells.

[53] Shi Y., Liu Z., Shen Y., Zhu H. (2018). A novel perspective linkage between kidney function and alzheimer’s disease. Front. Cell. Neurosci..

[54] Han S.W., Park Y.H., Jang E.S., Nho K., Kim S. (2022). Implications of liver enzymes in the pathogenesis of alzheimer’s disease. J. Alzheimers Dis..

[55] Harciarek M., Williamson J.B., Biedunkiewicz B., Lichodziejewska-Niemierko M., Dębska-Ślizień A., Rutkowski B. (2012). Risk factors for selective cognitive decline in dialyzed patients with end-stage renal disease: Evidence from verbal fluency analysis. J. Int. Neuropsychol. Soc..

[56] Liao W., Xu J., Li B., Ruan Y., Li T., Liu J. (2021). Deciphering the Roles of Metformin in Alzheimer’s Disease: A Snapshot. Front. Pharmacol..

[57] Mishra S., Prusty S.K., Sahu P.K., Das D.J. (2022). Irbesartan protects against aluminium chloride induced amyloidogenesis and cognitive impairment. J. Krishna Inst. Med. Sci..

[58] Sushko V.V., Sushko V.V. (2022). Use Acetazolamide in the Complex Therapy of Alzheimer’s Disease. Alzheimer’s Dement..

[59] Sberna G., Saez-Valero J., Beyreuther K., Masters C.L., Small D.H. (1997). The amyloid beta-protein of Alzheimer’s disease increases acetylcholinesterase expression by increasing intracellular calcium in embryonal carcinoma P19 cells. J. Neurochem..

[60] Umukoro S., Bakre T.O., Onwuchekwa C. (2010). Anti-psychotic and sedative effect of calcium channel blockers in mice. Afr. J. Med. Med. Sci..

[61] Stuve O., Weideman R.A., McMahan D.M., Jacob D.A., Little B.B. (2020). Diclofenac reduces the risk of Alzheimer’s disease: A pilot analysis of NSAIDs in two US veteran populations. Ther. Adv. Neurol. Disord..

[62] Yin C., Zhao R., Qian B., Lv X., Zhang P. Domain knowledge guided deep learning with electronic health records. Proceedings of the IEEE International Conference on Data Mining (ICDM).

[63] Su C., Xu Z., Pathak J., Wang F. (2020). Deep learning in mental health outcome research: A scoping review. Transl. Psychiatry.

[64] Zehraoui F., Sendi N., Abchiche-Mimouni N. (2022). MS-LSTMEA: Predicting clinical events for Hypertension using Multi-Sources LSTM Explainable Approach. SSRN 4123459.

[65] Zhang J. (2017). Representation Learning of Longitudinal Electronic Health Record Data for Patient Characterization and Prediction of Health Outcomes. Ph.D. Thesis.

[66] Visweswaran S., McLay B., Cappella N., Morris M., Milnes J.T., Reis S.E., Silverstein J.C., Becich M.J. (2022). An atomic approach to the design and implementation of a research data warehouse. J. Am. Med. Inform. Assoc..

[67] Rao S., Li Y., Ramakrishnan R., Hassaine A., Canoy D., Zhu Y., Salimi-Khorshidi G., Rahimi K. (2020). BEHRT-HF: An interpretable transformer-based, deep learning model for prediction of incident heart failure. Eur. Heart J..

[68] Altman D.G., Bland J.M. (2011). How to obtain the P value from a confidence interval. BMJ.

[69] Bonferroni C. (1936). Teoria Statistica delle Classi e Calcolo delle Probabilita.

