# Supplementary material for: Unveiling the Enigma: Exploring Risk Factors and Mechanisms for Psychotic Symptoms in Alzheimer’s Disease through Electronic Medical Records with Deep Learning Models
Source: Pharmaceuticals (Basel). 2023 Jun 21;16(7):911. doi: 10.3390/ph16070911 (PMC10385983; doi:10.3390/ph16070911)
Supplement: Supplementary file 1 [file pharmaceuticals-16-00911-s001.zip › pharmaceuticals-2406048-supplementary.pdf]

List S1. Diagnosis used for the identification of Alzheimer's disease.

1. Alzheimer's disease
2. early-onset Alzheimer's disease
3. late-onset Alzheimer's disease
4. Alzheimer's disease, unspecified

List S2. Diagnosis used for the identification of psychosis.

5. Unspecified psychosis
6. Senile dementia with delusional features
7. Hallucinations
8. Presenile dementia with delusional features
9. Delusional disorder
10. Depressive type psychosis
11. Other and unspecified reactive psychosis
12. Psychotic disorder with delusions in conditions classified elsewhere
13. Psychotic disorder with hallucinations in conditions classified elsewhere
14. Vascular dementia with delusions
15. Delusional disorders
16. Excitative type psychosis
17. Unspecified psychosis not due to a substance or known physiological condition
18. Hallucinations unspecified
19. Visual hallucinations
20. Psychotic disorder with hallucinations due to known physiological condition
21. Auditory hallucinations
22. Other hallucinations
23. Psychotic disorder with delusions due to known physiological condition
24. Psychogenic paranoid psychosis

List S3. Diagnosis used for the identification of delirium disorder.

1. Delirium due to conditions classified elsewhere
2. Vascular dementia with delirium
3. Senile dementia with delirium
4. Subacute delirium
5. Delirium due to known physiological condition
